# Dendritic Cells and Cancer: From Biology to Therapeutic Intervention

**DOI:** 10.3390/cancers11040521

**Published:** 2019-04-11

**Authors:** Ben Wylie, Christophe Macri, Justine D. Mintern, Jason Waithman

**Affiliations:** 1 Phylogica, Harry Perkins Institute, QEII Medical Centre, Nedlands, WA 6009, Australia; b.c.wylie@gmail.com; 2 Department of Biochemistry and Molecular Biology, The University of Melbourne, Bio21, Molecular Science and Biotechnology Institute, Parkville, VIC 3010, Australia; cmacri@unimelb.edu.au (C.M.); jmintern@unimelb.edu.au (J.D.M.); 3 Telethon Kids Institute, University of Western Australia, Northern Entrance, Perth Children’s Hospital, Nedlands, WA 6009, Australia

**Keywords:** tumor-associated dendritic cells, DC-based therapy, immunotherapy, checkpoint blockade, immunosuppression, novel therapies, cancer

## Abstract

Inducing effective anti-tumor immunity has become a major therapeutic strategy against cancer. Dendritic cells (DC) are a heterogenous population of antigen presenting cells that infiltrate tumors. While DC play a critical role in the priming and maintenance of local immunity, their functions are often diminished, or suppressed, by factors encountered in the tumor microenvironment. Furthermore, DC populations with immunosuppressive activities are also recruited to tumors, limiting T cell infiltration and promoting tumor growth. Anti-cancer therapies can impact the function of tumor-associated DC and/or alter their phenotype. Therefore, the design of effective anti-cancer therapies for clinical translation should consider how best to boost tumor-associated DC function to drive anti-tumor immunity. In this review, we discuss the different subsets of tumor-infiltrating DC and their role in anti-tumor immunity. Moreover, we describe strategies to enhance DC function within tumors and harness these cells for effective tumor immunotherapy.

## 1. Introduction

Dendritic cells (DC) represent a heterogenous group of innate immune cells that infiltrate tumors and process and present tumor-derived antigens to naïve T cells. DC play a critical role in priming anti-tumor T cell immunity and thereby represent a major therapeutic target for cancer immunotherapy. The anti-tumor function of DC can be impeded by suppressive signals present in the tumor microenvironment. In addition, DC with immunosuppressive activity can be recruited to tumors, eliciting T cell tolerance and progressive tumor growth. Developing novel DC-targeted therapies is important to exploit the capacity of DC to initiate and enhance effective anti-tumor immunity. In this review, we first discuss the biology of tumor-associated DC by detailing the DC subsets present in tumors. We then address anti-cancer strategies and examine how these therapeutic interventions impact tumor-associated DC function.

## 2. DC Subsets in Cancer

**Figure 1:**
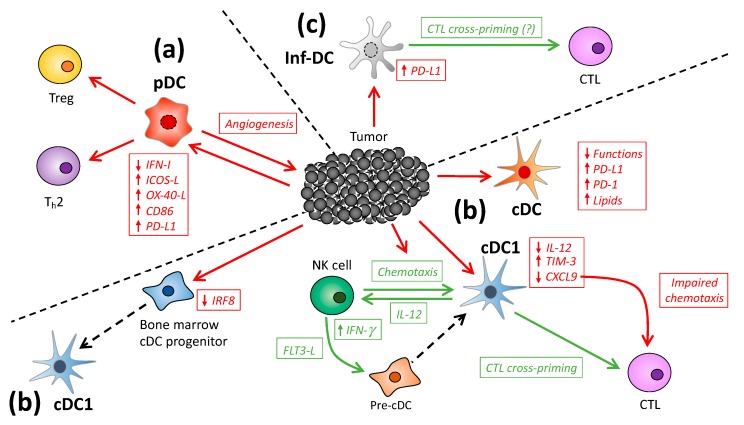
The biology of DC in the tumor microenvironment: (**a**) pDC; (**b**) cDC; and (**c**) inf-DC subsets infiltrate the tumor microenvironment and either support the anti-tumor immune response or promote tumorigenesis. Tumors frequently develop strategies to alter DC development, tumor infiltration and function. The mechanisms that promote anti-tumor immunity are shown in green, while those that act to promote tumorigenesis are displayed in red.

The DC family encompasses multiple DC subsets with specific immune functions that are highly conserved between mouse and humans (summarized in Table 1). During cancer, the different DC subtypes are localized in and/or recruited to tumors. Here, we discuss their role during cancer and particularly describe how, on the one hand, tumor DC can act to elicit enhanced anti-tumor immunity, while, on the other hand, these cells can be subjected to suppressive mechanisms that ultimately promote tumorigenesis (Figure 1).

### 2.1. Plasmacytoid DC

Plasmacytoid DC (pDC) are recognized as major producers of type I interferons (IFN-I) and act to orchestrate immunity against viral infections. In settings of cancer, pDC derived IFN-I can promote anti-tumoral immunity through its direct activity on both tumor and immune cells [1]. pDC also secrete an array of other inflammatory cytokines and chemokines and can act as antigen presenting cells, however with lower efficiency than conventional DC (cDC) [2]. Antigen presentation by pDC is largely considered to evoke tolerance and the induction of T cell anergy and/or deletion. This is due to the capacity of pDC to secrete tolerogenic factors such as interleukin (IL)-10, tumor-growth factor (TGF)-β and indoleamine 2,3-dioxygenase (IDO). Furthermore, pDC can engage inhibitory receptors on T cells by expressing several of their ligands, including inducible T-cell costimulatory ligand (ICOS-L), OX40 ligand (OX40-L) and programmed cell death ligand 1 (PD-L1) [3].

Numerous studies have identified pDC infiltration in several different type of tumors, with their presence often being predictive of a poor prognosis. In ovarian cancer, for example, pDC accumulate in the tumor epithelium, but not in the ascites, and this is associated with early relapse [4,5]. High numbers of pDC are present in skin lesions and draining lymph nodes of melanoma patients [6] and in breast tumor biopsies [7], with a strong correlation between the presence of pDC and tumor aggressiveness, poor clinical outcomes and shorter survival. It is well established that tumor-infiltrating pDC are poorly immunogenic and have a significantly impaired capacity to produce IFN-I [4,7,8,9,10]. This dysfunction is mostly limited to pDC that are localized to the tumor microenvironment. In contrast, the pDC isolated from the tumor-draining lymph nodes in different type of cancers remain fully competent in IFN-I production [8,11]. Factors produced locally by tumor cells have been implicated in altering the IFN-I expression in pDC. In mice carrying B16 and TC1 tumors, TGF-β has been identified as the major tolerogenic cytokine produced by tumor cells that is responsible for this suppression [12]. Head and neck tumors reduce the IFN-I-producing capacity of pDC by releasing both TGF-β and prostaglandin E2 (PGE_2_), a bioactive lipid with immunosuppressive properties [13]. In line with this study, mice inoculated with PGE_2_-producing BRAFV600E melanoma cells show reduced expression of several IFN-stimulated genes at the tumor site. This may indicate that melanoma-derived PGE_2_ exerts an inhibitory effect on IFN-I production by tumor-associated pDC, thus altering the downstream gene signature [14]. In breast cancer patients, tumor-derived TGF-β and tumor necrosis factor (TNF)-α act synergistically on pDC to inhibit their IFN-I expression by downregulating the IFN regulatory factor (IRF) 7 [15]. IL-10 produced by tumor-infiltrating myeloid cell and Foxp3^+^ CD4^+^ regulatory T cells (Treg) is a major tolerogenic cytokine that downregulates IFN-I expression in pDC. Such inhibition has been observed when pDC were incubated with conditioned medium from head and neck carcinoma and this effect was partially reversed in the presence of anti-IL-10 blocking antibodies [9]. Conversely, in multiple myeloma, downregulation of IFN-I production by pDC is caused by cell-to-cell contact between pDC and tumor cells. This process activates E-cadherin on pDC, leading to the expression and activation of the ubiquitin-editing enzyme A20 that regulates Toll-like receptor (TLR)-9 ubiquitination. As a result, TLR-9 is targeted for degradation and can no longer elicit signals required for the induction of IFN-I expression [16]. 

While the immunogenicity of tumor-associated pDC is impaired, their tolerogenic functions are frequently harnessed by tumor cells to foster tumor growth. The cytokines that pDC produce may be harnessed by the tumorigenic process. For example, pDC recruited by ovarian tumor ascites support tumor vascularization through the secretion of angiogenic cytokines TNF-α and IL-8 [17]. Furthermore, the capacity of pDC to induce T cell tolerance can promote tumor expansion. Wei et al. have shown that the co-culture of pDC from ovarian tumors with allogenic CD8^+^ T cells results in the generation of IL-10-producing-CD8^+^ regulatory T cells with strong immunosuppressive properties [18]. Consistent with this, tumor-associated pDC expand Treg populations within the tumor. This process has been linked to pDC impaired IFN-I secretory function [7] and to high surface levels of ICOS-L. Indeed, Treg in humans encompass two populations based on the expression of the receptor ICOS [19]. Several authors have proposed that tumor-associated pDC provide a co-stimulation signal and induce Treg expansion through ICOS-L-ICOS interaction. This mechanism may explain the striking accumulation of ICOS^+^ Treg observed in ovarian, breast and gastric tumor tissues [20,21,22]. Similarly, pDC purified from liver tumor tissues enhance IL-10 secretion by CD4^+^ Treg via an ICOS-dependent mechanism [23]. In human melanoma, ICOS-L together with the immune checkpoint ligand OX40-L, are highly expressed by pDC and drive the polarization of allogenic CD4^+^ T cells into T helper cell (Th) 2 and Treg-like cells [6]. Other pDC ligands involved in T cell tolerance induction may also play a role in cancer progression. In patients with chronic myeloid leukemia, high pDC expression of CD86, which activates the T cell inhibitory receptor CTLA4, is a strong predictive marker for relapse [24]. Another example is PD-L1, which provides a negative signal to T cells by interacting with the receptor PD-1. The blocking of PD-L1 on pDC purified from multiple myeloma patients results in enhanced T cell activation in allogenic co-cultures. This suggests that pDC are able to suppress T cells during cancer progression through PD-1 engagement [25].

### 2.2. Conventional DC

Conventional DC (cDC) can be subdivided into two populations in both mice and humans: cDC1 and cDC2. Mouse cDC1 include most lymphoid-resident CD8^+^ DC and the tissue resident and migratory CD103^+^ DC. Human cDC1 are defined as CD141^+^ DC [26]. Both mouse and human cDC1 specifically express the surface markers Clec9A [27,28] and XCR1 [29,30] and require the transcription factors Basic Leucine Zipper ATF-Like Transcription Factor 3 (BATF3), IRF8 and ID2 for their development [31]. cDC2 are classified as CD11b^+^ DC in mice and CD1c^+^ DC in humans [26] and are developmentally dependent on the transcription factors IRF4 and Zinc finger E box binding homeobox 2 (ZEB2) [31,32]. Interestingly, a new human DC subset expressing Axl and Siglec6 has recently been identified by single-cell RNA sequencing and cytometry by time-of-flight (CyTOF) [33,34,35]. Although Axl^+^ DC cluster near pDC [33,34], they differentiate into cDC-like cells when cultured with stromal cells and DC hematopoietins, suggesting they may be cDC precursors [34]. Functional analyses also highlight a close relationship between Axl^+^ DC and cDC [33,35]. 

cDC are the most potent antigen-presenting cells and, as such, are strong inducers of T cell-mediated immune responses. Mouse cDC1 are highly efficient in antigen cross-presentation, i.e., the presentation of extracellular antigens by MHC I molecules, and therefore are critical at priming CD8^+^ T cell responses to exogenous tumor antigen [36]. Mouse cDC1 are strong producers of IL-12 that drive the polarization of activated CD8^+^ T cells into cytotoxic T lymphocytes (CTL) [37]. In contrast, mouse cDC2 are specialized in MHC II presentation and the induction of CD4^+^ T cell responses, including Th1, Th2 and Th17 cells [26]. In humans, there is not a clear segregation of antigen presentation activities between the major cDC subsets, as is the case in the mouse. While human cDC1 are superior at cross-presentation of cell-associated antigens, both cDC1 and cDC2 have been shown to cross-present soluble antigens at comparable levels. Human cDC1 and cDC2 are equally competent at MHC II presentation and CD4+ T cell priming [26]. Human Axl^+^ DC are efficient stimulators of CD4^+^ and CD8^+^ T cells in allogenic cultures [33,35], however the antigen presentation pathways utilized by these DC have not been studied.

cDC subsets infiltrate tumors in mice and humans, although they constitute a very minor population of all intratumoral immune cells. For example, CD103^+^ cDC1 and CD11b^+^ cDC2 subsets are present at low frequencies in mouse models of melanoma [38,39,40,41], lung and colon carcinoma [39,42] and mammary tumors [42,43]. Both cDC subsets are recruited to tumors as pre-cDC precursors, similar to cDC that reside in lymphoid organs and other peripheral tissues. Tumor-associated pre-cDC exhibit the same phenotypic and functional characteristics as bone-marrow and spleen pre-cDC and their migration depends on tumor expression of chemokine CCL13. Once recruited, pre-cDC proliferate and differentiate into competent cDC [44]. In cancer patients, cDC1 and cDC2 have been detected in melanoma [42,45], breast cancer [42,46], non-small cell lung carcinoma and colorectal cancer [42]. Furthermore, a recent single cell analysis of early lung adenocarcinoma revealed the presence of two clusters in the tumor tissues, corresponding to cDC1 and cDC2 subsets [47]. Despite their paucity, the tumor content of cDC, and more specifically in cDC1, is a reliable predictor of a good prognosis for cancer patients. Transcriptomic analysis of different human tumors, including melanoma and breast cancers, reveals that the cDC1-specific signature correlates with overall patient survival [38,39,46]. Furthermore, Ruffel et al. showed that, during breast cancer, expression of cDC1-specific cytokine IL-12 significantly correlates with an increased response to chemotherapy [43].

cDC1 are critical to initiate CTL immunity against tumors [48,49,50]. Using mice inoculated with fluorescent tumors, several researchers have demonstrated that antigens are transported from the tumor site to the tumor-draining lymph node by migratory CD103^+^ cDC1. This migration requires expression of homing chemokine receptor CCR7. Once in the lymph node, CD103^+^ cDC1 activate naïve CD8^+^ T cells with evidence suggesting that tumor antigens are also simultaneously transferred to the lymphoid resident CD8^+^ cDC1 and CD11b^+^ cDC2 [41,49,50]. Consistent with this finding, the induction of a CTL immune response in the tumor draining lymph nodes is abolished in Zbtb46-DTR–transgenic mice that do not develop cDC [40] and in Sec22b^-/-^ mice that possess cDC1 deficient in cross-presentation [51].

cDC1 can shape the immune response within tumors. Spranger et al. showed that CTL are absent from tumors of cDC1-deficient BATF3^-/-^ mice. This occurs because tumor-associated cDC1 are the major source of tumoral CXCL10 that acts as a chemoattractant for CTL that express the chemokine receptor CXCR3 [52]. cDC1 also establish cross-talk with natural killer (NK) cells in tumors to regulate local immunity. Tumor NK cells produce chemokines XCL1 and CCL5 that bind to receptors expressed by cDC1 driving their accumulation in tumors [38]. Pre-cDC1 precursors do not express XCR1 and CCR5 [53], implying that NK cells chemoattract a fully differentiated population of cDC1. NK cells are also able to promote cDC1 development in situ by producing the DC hematopoietin Fms-like tyrosine kinase-3 ligand (Flt3-L) [45]. On the other hand, tumor-associated cDC1 regulate NK cells by supporting their production of IFN-γ in an IL-12-dependent manner. This plays an important role in the NK cell control of tumor metastasis [54].

cDC are a target for tumor immunosuppressive mechanisms that impair their development, tumor access and/or function. For example, tumor-derived granulocyte-colony stimulating factor (G-CSF) during breast and pancreatic cancer inhibits the hematopoiesis of cDC1 in mouse and human bone-marrow. This immunosuppression involves IRF8 downregulation in bone-marrow cDC precursors by G-CSF, thereby altering cDC1 differentiation [55]. Migration of cDC1 is also reduced when PGE_2_ is expressed by tumor cells. In this case, PGE_2_ disrupts the NK-cDC1 chemotactic axis by downregulating expression of chemokines XCL1 and CCL5 in NK cells and their receptors XCR1, CCR1 and CCR5 on cDC1 [38]. In patients with early lung adenocarcinoma, the presence of cDC1, but not cDC2, is strongly reduced in tumors compared to the non-involved lung tissues from the same patients, thus supporting a similar mechanism of cDC1 exclusion [47]. Tumor-derived inhibitory molecules can also impact cDC function. For example, TGF-β and vascular endothelial growth factor (VEGF) produced by B16 melanoma have an inhibitory effect on the activation, cytokine expression and T cell stimulatory capacities of cDC [56]. Similarly, macrophages that infiltrate breast tumors are strong producers of IL-10. This cytokine suppresses cDC1 expression of IL-12, and thereby inhibits the downstream anti-tumor CD8^+^ T cell response [43]. Another cause of cDC dysfunction during cancer is the alteration of their overall lipid content. Herber et al. reported that mouse and human cDC localized in tumors have an unusually high triglyceride content. This is due to the upregulation of macrophage scavenger receptor 1 that is involved in intracellular transport of lipids and therefore increases lipid uptake by cDC. Alteration in lipid content interferes with MHC I and MHC II presentation by impairing antigen degradation in endosomes, however the exact molecular mechanism responsible remains to be elucidated [57]. Tumor-associated cDC contain lipid bodies with oxidatively truncated electrophilic lipids. These modified lipids covalently interact with the chaperone Hsp70 leading to impaired MHC I-peptide trafficking and reduced CD8^+^ T cell activation [58].

Although inhibitory immune checkpoint receptors are classically viewed as molecules associated with T cell exhaustion [59]; some members of this family are also expressed by cDC and further contribute to an impairment in DC function during cancer. In hepatocellular carcinoma, both mouse and human tumor-infiltrating cDC express PD-1 on their surface and in this setting, PD-1 acts as a brake on cDC by limiting their T cell stimulation capacities [60]. De Mingo Pulido et al. identified T-cell immunoglobulin and mucin-domain containing-3 (TIM-3) as another inhibitory immune checkpoint receptor that is highly expressed by cDC that infiltrate breast tumors. TIM-3 provides an inhibitory signal that downregulates the chemokine CXCL9. Consequently, CTL expressing the CXCL9 receptor CXCR3 are excluded from tumors [61].

### 2.3. Inflammatory DC

Inflammatory DC (inf-DC), also referred to as monocyte-derived DC (moDC), are absent from steady-state tissues and differentiate from monocytes during inflammation, infection and cancer [42,62,63]. Their phenotype in mice is characterized by the expression of several macrophage markers such as F4/80, Ly6C, CD64 and FcεR1 [64]. Their human homologous have been identified in several tissues and lymphoid organs from patients with inflammatory diseases, but also in tissues from healthy donors [65]. Based on their gene signature, human inf-DC are considered the in vivo equivalents of in vitro-derived moDC that have been extensively used in DC research and in clinical settings for vaccination [66]. Mouse inf-DC can cross-present antigens to CD8^+^ T cells [67,68] and are able to induce Th1 and Th2 responses depending on the inflammatory conditions [69,70]. Humans inf-DC undertake cross-presentation [71] and are potent inducers of Th17 in cocultures with allogenic CD4^+^ T cells [66].

The presence of inf-DC in tumors has not been well characterized. Reports have shown that circulating monocytes are chemoattracted to tumors via CCL2 [72,73,74], thereby suggesting that infiltrating monocytes may develop in situ into inf-DC. Tumor-associated inf-DC have been detected in several mouse cancer models and in patients with non-small cell lung carcinoma and colorectal cancer [42]. A population of CD1c^+^ CD14^+^ DC that may be related to inf-DC have been detected in skin lesions of melanoma patients [75]. The role of inf-DC during cancer is not well defined, with limited studies reporting conflicting results. In mice with B16 melanoma, inf-DC from tumors efficiently cross-present tumor antigens and are able to induce strong CTL proliferation, suggesting that inf-DC may promote tumor-specific CD8^+^ T cell immune responses [40]. This is consistent with the identification by Sharma et al., of a new population of cross-presenting DC with anti-tumor activity in inflamed tumors. These DC express Ly6C and arise from the monocytic lineage, thus indicating a strong resemblance with inf-DC. These cells, however, also display some hallmark cDC1 characteristics including the surface expression of CD103 and BATF3 dependency for their development. Hence it is unclear whether this DC population can be classified as bona-fide inf-DC [76]. In contrast, other studies support a tumorigenic role of inf-DC. Tumor-associated inf-DC from mice developing lung carcinoma actively inhibit CD4^+^ and CD8^+^ T cell responses by releasing nitric oxide [42], a reactive radical with known T cell suppressive activities [77]. Furthermore, the CD1c^+^ CD14^+^ inf-DC-like populations that accumulate in melanoma nodules are hypostimulatory for allogenic T cells and have high cell surface expression of PD-L1. Blockade of PD-L1 partially reduces this inhibition, thus highlighting a PD-1-dependent T cell suppression mechanism [75]. In line with this, inf-DC generated in vitro in the presence of prostate carcinoma stromal factors also display high PD-L1 expression and low T cell stimulation capacities [78].

## 3. Tumor-Associated DC and Cancer Therapy

**Figure 2:**
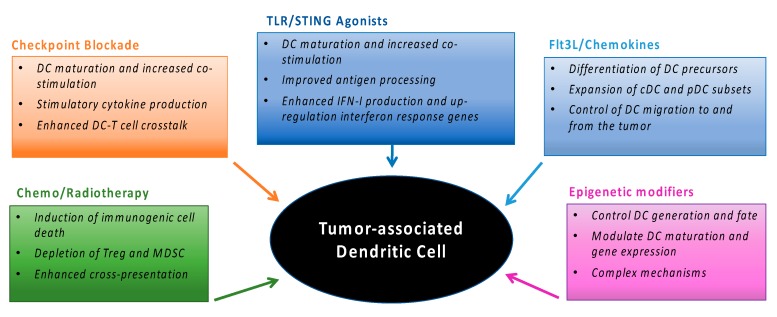
Therapies to modulate tumor-associated DC and enhance anti-tumor immunity. Different interventions can alter the development, maturation, migration and/or function of tumor associated DC.

An emerging concept in the field of cancer therapy is the recognition that many clinically effective treatments have an immune-mediated anti-tumor component. Most new anti-cancer immunotherapies are trialed in combination with conventional therapies including chemotherapy and radiotherapy. How these therapies impact DC is not well understood. However, it is becoming increasingly clear that many anti-cancer therapies alter tumor-associated DC function (Figure 2). Here, we discuss the impact that anti-cancer therapies have on DC and how this knowledge can be exploited to boost DC activity against tumors. 

### 3.1. Current Cancer Therapies and DC

Chemotherapy enhances anti-tumor DC function through multiple mechanisms that include induction of immunogenic cell death (ICD), increased antigen availability and/or depletion of suppressive immune cell subsets [79]. For example, the effectiveness of anthracyclines is dependent on the recruitment and maturation of DC within the tumor [80]. Anthracyclines induce ICD, resulting in exposure of calreticulin on the surface of tumor cells [81], tumor secretion of high mobility group box 1 [82] and activation of NLR Family Pyrin Domain Containing 3 (NLRP3) inflammasome in DC [83]. Other chemotherapeutic agents such as cyclophosphamide and platinum-based compounds not only induce ICD, but also enhance DC activity by depleting suppressive Treg within tumors [84,85]. Similar mechanisms are observed following gemcitabine treatment, which results in a reduction of myeloid-derived suppressor cells (MDSC) [86]. Chemotherapies also act directly to enhance the activation and function of tumor-associated DC. McDonnell et al. showed that systemic gemcitabine therapy restores the capacity of suppressed or immature-like tumor-infiltrating DC to cross-present antigens [87]. DC exposed to 5-fluorouracil increase IL-12 production and present tumor-associated antigens with increased efficiency [88]. This response is dependent on DC expression of the formyl peptide receptor 1 [89]. The release of immune-stimulatory proteins from tumors during cisplatin therapy increases DC recruitment to tumors and upregulates DC co-stimulatory molecules CD80/86 and CD70 [90].

Radiotherapy, similar to chemotherapy, also induces tumor cell destruction and ICD (reviewed by [91]). Interestingly, localized radiotherapy can promote immune-mediated tumor clearance at sites distant to the irradiated area, a phenomenon known as the “abscopal effect” [92,93]. Importantly, the regime of radiotherapy appears to be critical for this response as fractionated, but not single high dose, radiation is reported to promote systemic anti-tumor responses [94]. This may be due to the induction of the DNA exonuclease Trex1 in cancer cells by high, but not low dose irradiation, which facilitates the degradation of cytosolic DNA [95]. Activation of Trex1 leads to attenuated signaling through the stimulator of IFN genes (STING) pathway and decreased IFN-β production, resulting in a lack of cDC1 recruitment to the tumor [95]. This lack of cDC1 migration likely stems from limited upregulation of chemoattractant CCL5 [38]. Results from recent preclinical trials investigating the use of radiotherapy in combination with immunotherapy have revealed the mechanistic importance of radiation dose, fractionation and timing [96,97]. Multiple reports exist demonstrating radiotherapy synergizes with DC-based therapies, including the administration of Flt3-L to expand cDC1 in vivo [98] and injection of ex vivo generated DC directly into the tumor [99,100]. This is further supported by a recent phase I clinical trial where stereotactic ablative radiotherapy enhanced the anti-tumor effect of a combined DC vaccine and immune-stimulatory adjuvant cocktail, with five out of six patients receiving targeted radiotherapy presenting with stable disease [101]. These data suggest that combining DC-targeted therapeutics with radiotherapy is an effective strategy to prime anti-tumor immunity.

Tumor-associated DC function is important in the response to checkpoint blockade immunotherapy. Successful outcomes following anti-PD-1 therapy require DC-T cell crosstalk [102] and the initiation of anti-tumor CD8^+^ T cell responses by Sec22b-dependent cross-presentation in DCs [51]. Targeting novel checkpoint ligands on DC that modulate T cell activity is an attractive strategy for immunotherapy. Lymphocyte activation gene-3 (LAG-3) is expressed on activated T cells and regulatory pDC within tumors and modulates pDC homeostasis and IFN-I production after TLR9 stimulation [103,104]. Treatment with an anti-LAG-3 antibody promotes human moDC maturation in ex vivo cultures with significant up-regulation of co-stimulatory molecules and enhanced production of IL-12 and TNF-α [105]. In a clinical trial for metastatic breast cancer, a soluble LAG-3-Ig fusion protein was used in combination with paclitaxel chemotherapy. Increased activation of antigen-presenting cells and concurrent expansion of effector-memory CD8^+^ T cells and NK cells was observed, correlating with favorable response rates relative to controls [106]. This highlights LAG-3-targeted therapies as a viable immunogenic platform that can be combined with chemotherapy.

TIM-3 is an inhibitory immune checkpoint that is highly expressed by tumor-associated DC. Treatment with an anti-TIM3 monoclonal antibody enhances CXCL9 expression by CD103^+^ cDC1, leading to increased CTL recruitment to the tumor [61]. In clinical settings, anti-TIM-3 antibody treatment improves responses when delivered in combination with Paclitaxel [61] and the immune checkpoint antibodies anti-CTLA4 and anti-PD-1 [107], resulting in the regression of established tumors. TIM-3 therefore has potential as a novel immune checkpoint, which can be targeted to improve the function of tumor-associated DC. V-domain Ig suppressor of T cell activation (VISTA) is another novel checkpoint ligand that is highly expressed on DC and directly suppresses T cell proliferation [108]. A combination regimen of an anti-VISTA blocking antibody administered with a cancer vaccine and TLR adjuvant demonstrated synergistic efficacy and impaired growth of established B16 melanoma [109]. Blocking VISTA on DC results in increased T cell recruitment to the tumor microenvironment and enhances CD8^+^ T cell activation by converting resting and exhausted cells into functional effector cells. VISTA blockade is particularly effective in combination with anti-CTLA4 antibody therapy [110], which likely results from increased VISTA expression by myeloid cells after anti-CTLA4 antibody therapy [111]. Similarly, the synergistic potential of anti-VISTA and anti-PD-1 antibody therapy has been suggested, given they induce suppressive signaling via independent pathways [112]. Therefore, evidence implicates VISTA as a novel modulator of DC function with blockade of VISTA a potential treatment modality for stimulating anti-tumor immunity. Efficacy and safety profiles of VISTA-targeted therapy as a fully human monoclonal antibody or oral inhibitor are currently being explored in Phase I clinical trials of advanced solid tumors and lymphoma (reviewed in [113]).

Cancer therapeutics that inhibit critical kinases required for tumor cell growth are also reported to have immunomodulatory effects on DC. The tyrosinase kinase inhibitor (TKI) sunitinib decreases suppressive immune subsets in the tumor microenvironment and down-regulates PD-L1 expression on tumor-associated myeloid cells and pDC. In combination with IL-12 and 4-1BB-L, sunitinib improves long-term survival of mice bearing large MCA26 tumors [114]. In conflict with this, other studies have shown that the TKIs sorafenib and imatinib inhibit DC function, and the differentiation of CD34^+^ DC progenitors in vitro [115]. DC progenitors express lower levels of co-stimulatory molecules CD80 and CD40 after culture with imatinib and have attenuated T cell stimulatory capacity [116]. In light of these findings, the combination of TKIs with DC-based therapies should be carefully considered. BRAF inhibitors, commonly used to treat melanoma, are also reported to exert pro-inflammatory effects on DC including inflammasome activation, increased MHC expression and increased IL-1β production [117]. Combining BRAF inhibition therapy with Flt3-L and the TLR-3 agonist polyinosinic:polycytidylic acid (poly-I:C), to expand and activate intra-tumoral cDC1, leads to more effective anti-tumor therapy [41]. The synergistic potential of BRAF inhibitors and immune-based therapies was well reviewed by Reddy et al. [118]. 

### 3.2. Enhancing DC Activation during Cancer Therapy 

One goal of DC-targeted therapies is to enhance tumor-associated DC function and drive effector T cell recruitment into the tumor. As tumor-associated DC are often functionally impaired it is critical to develop adjunct therapies that facilitate DC activation and maturation. TLR ligation is necessary during antigen encounter for DC maturation and upregulation of co-stimulatory molecules [119], without which DC can become regulatory or tolerogenic [120]. TLR signaling also influences how DC process and present antigens [121]. Numerous TLR agonists have been investigated in clinical trials in combination with DC vaccination and other immunotherapies [122,123]. Of these agents, the TLR3 agonist poly-I:C and its more stable derivatives such as poly-ICLC [124] are particularly favorable candidates. TLR3 is highly expressed by cross-presenting DC subsets, which are critical to induce anti-tumor CD8^+^ T cell responses [125]. Treatment of ovarian cancer patients with poly-ICLC in combination with a synthetic long peptide vaccine specific for the tumor-associated antigen NY-ESO-1 results in elevated antigen-specific antibody titers and increased T cell responses [126]. Similar results have been obtained after poly-ICLC administration and vaccination against a combination of tumor-associated antigens in low grade glioma [127]. Poly-I:C activation of DC via TLR3 results in IFN-III (IFNλ) production by cDC1 via IRF3 and IRF7 activation independent of MyD88 [128], while poly-I:C signaling via Melanoma Differentiation-Associated protein 5 (MDA-5) strongly induces IFN-I production [129], which promotes DC maturation and Th1 type CD4^+^ T cell immunity. Importantly, both receptors have distinct and complementary functions that are critical for the activation of anti-tumor immunity by cDC1 [130,131]. Therefore, this should be considered in the design of novel poly-I:C based adjuvants. Other TLR agonists may also be beneficial for DC-mediated anti-tumor immunity. The TLR7 agonist Imiquimod has been shown to enhance immune-mediated tumor regression in melanoma, basal cell carcinoma and breast cancer when applied as a topical cream (Aldara) [132,133,134]. Topical delivery to the site of the tumor suggests the mode of action is primarily the maturation of tumor-associated DC, particularly pDC, which express high levels of TLR7. Activation of DC via TLR7 leads to increased IFN-I signaling as well as enhanced antigen presentation and T cell priming within the tumor [135]. Similarly, the TLR9 agonist CpG-oligodeoxynucleotide (CpG-ODN) elicits anti-tumor activity through the induction of IFN-I in pDC [136]. TLR9 activation signals via recruitment of MyD88 to activate IRF7 [137], resulting in the release of IFN-I, which is critical for immune-surveillance of tumors [138]. CpG-ODN-based TLR agonists have demonstrated the potential to enhance tumor eradication and T cell responses in combination with checkpoint blockade therapy and DC vaccination [139,140]. Therefore, pDC represent good candidates for therapy targeting tumor-associated DC to boost anti-tumor immunity. pDC dysfunction in the tumor microenvironment, however, remains a barrier to successful therapy and it is reported in some solid tumor models that intra-tumoral administration of CpG fails to reprogram the tolerogenic phenotype of tumor-associated pDC [141].

An approach to boost DC function in the context of anti-tumor vaccination is to elicit DC activation via enhanced co-stimulatory molecule expression. One strategy involves ex vivo activation of DC using the mRNA transfection-based delivery of a cocktail of co-stimulatory molecules (including CD40L, CD70, and constitutively active TLR-4) called TriMix [142]. DC activated by TriMix potently induce the expansion of functional MelanA-specific CD8^+^ T cells [143]. DC exposed to TriMix activate CD4^+^ and CD8^+^ T cells, promote their secretion of IFNγ and reduce the suppressive potential of Treg, partially alleviating Treg inhibition of CD8^+^ T cell immunity [144]. Delivery of TriMix DC in melanoma patients triggers durable tumor responses in late stage disease [145], suggesting this could be a novel DC-targeted strategy. To avoid culturing DC ex vivo Van Lint et al. delivered tumor antigen and TriMix encoding-RNA via intranodal injection. In several mouse tumor models this delivery method was similarly effective as the delivery of electroporated DC [146]. Successful modulation of tumor-associated DC has also been achieved by direct intra-tumoral injection of TriMix. Tumor-associated DC are reprogrammed into mature antigen-presenting cells that promote systemic anti-tumor immunity after migration from the tumor to the draining lymph node [147].

Another target to enhance DC activation is STING, which can be triggered by intra-tumoral delivery of cyclic dinucleotides. Activation of the STING pathway controls tumor growth in mouse models of melanoma and breast cancer, mediating systemic immunity by enhancing the efficacy of anti-PD-1/CTLA 4 therapy [148] or in combination with OX40 receptor and PD-L 1 blockade [149]. Similar to TriMix, a vaccination strategy utilizing cyclic dinucleotides, to activate the STING pathway, in addition to GM-CSF-secreting tumors mediates regression of established melanoma [150] and enhances the efficacy of anti-PD-1 antibody therapy [151]. The mechanism of action was shown to be STING-dependent, and synergy with TLR agonists was reported, suggesting STING is a novel DC-targeted strategy with potential for combination with checkpoint blockade and DC vaccination [152].

The epigenetic landscape of tumors, including the immune cells present in the tumor microenvironment is dysregulated [153]. While the epigenetic control of DC is not well understood, particularly within the tumor, there is evidence that epigenetic modifications control DC function [154] and development [155]. Furthermore, it has been reported that TGFβ, a key immunosuppressive cytokine present in the tumor milieu, can induce chromatin modifications of H3K4me and H3K27 epigenetic marks that negatively impact DC differentiation and function [156]. Therefore, therapy that targets epigenetic dysregulation in tumor-associated DC may help restore DC function. A recent study of the effects of epigenetic modifiers on ex-vivo generated DC reported that two clinically relevant agents, given in combination with IFN-α 2, strongly induced IRF7 and IRF8 activation as well as expression of TLR3, TLR7 and IFN-β by DC [157]. These results suggest epigenetic modification is capable of skewing DC towards a more functional phenotype; however, whether this therapy will succeed in modulating tumor-associated DC in vivo remains to be determined. Epigenetic therapy does induce IFN-I and IFN-responsive genes in tumors via the induction of endogenous retroviruses silenced by DNA methylation [158], potentially augmenting DC maturation and function within the tumor. Clinical data suggest that epigenetic therapy can boost T cell responses generated by DC vaccination against the NY-ESO-1 antigen in myelodysplastic syndrome [159]. Therefore, the epigenetic modulation of DC remains a promising approach with the potential for enhancing DC function and anti-tumor immunity [160].

## 4. Modulating DC Generation and Migration During Cancer Therapy

Flt3-L treatment is a promising strategy to elicit anti-tumor CTL immunity. Flt3 is expressed at high levels by cDC1, a DC subset of major importance to anti-tumor immunity. cDC1 expand in number in response to in vivo administration of Flt3-L [161,162]. Combining Flt3-L and poly-I:C treatment induces expansion and activation of CD103^+^ cDC1 from DC progenitors within the tumor and enhances the efficacy of checkpoint therapy blockade in experimental models of melanoma [41] and glioblastoma [163]. In the context of DC vaccination, the addition of Flt3-L enhances the efficacy of RNA vaccination, with pDC rather than cDC1 essential for the adjuvant effect of Flt3-L in this setting [164]. Experimental vaccination strategies, such as those using Flt3-L expressing B16 melanoma cells, demonstrate higher levels of CD8^+^ T cell and DC infiltration compared to vaccination with GM-CSF-expressing tumor cells [165]. Supporting these findings, Flt3-L stimulation was shown to produce DC with lower inflammatory cytokine production and superior migration potential in vivo, compared to GM-CSF/IL-4 stimulated DC [166]. In clinical trials, recent results from a phase II study in human melanoma suggest that Flt3-L treatment efficiently mobilizes DC and enhances responses to DC-targeted vaccines [167]. Therefore, pre-treatment with Flt3-L assists the generation of cross-presenting cDC1 for CTL induction and enhances other DC-targeted therapies.

Chemokine-based therapy represents another strategy to drive immune cell infiltration into the tumor microenvironment. DC migration to the lymph node for T cell priming is guided by inflammatory chemokines CCL19, CCL20 and CCL21 mediated by CCR6 and CCR7 expression on DC [168]. As discussed above, NK cells are involved in the chemoattraction of cDC1 by secreting XCL1 and CCL5 [38,45]. Protective T-cell dependent anti-tumor immunity can be induced with the genetic fusion of chemokines to a self-tumor antigen [169]. A vaccine strategy based on XCL1- and IL-2-secreting neuroblastoma cells enhanced T-cell infiltration and resulted in complete or partial tumor remission in vaccinated patients [170]. Similarly, adenoviral delivery of XCL1 and tumor-associated antigens to DC increases IL-2 and IFNγ production by NK and T cell populations [171]. Enforcing local expression of XCR1 at the tumor site results in increased CD4^+^ T cell, CD8^+^ T cell and neutrophil infiltration and eradication of established tumors [22,172]. Other cytokines mediate DC trafficking to specific organs [173] suggesting that specific cytokine therapy may be applicable to direct DC migration towards a desired tissue. A screen of chemokine-transduced tumors identified CCL19, CCL22 and XCL1 as potent mediators of immune-based tumor rejection in vivo [174]. Notably, the context of chemokine therapy and the tumor microenvironment are important determinants of this response. For example, inflammatory chemokine CCL3, a potent DC attractant, drives DC and immune cell infiltration when expressed by B16 melanoma, but this does not lead to tumor regression [175]. While using chemokines is a potential strategy to enhance DC tumor infiltration, it may be important to overlay additional therapeutics to promote effective immunity at the tumor site.

## 5. Conclusions

The success of anti-cancer immunotherapy has placed DC under the spotlight, given their critical role in initiating anti-tumor T cell immunity. The complexity of DC subtypes, and their interactions, means that multiple complementary strategies are likely necessary to drive the eradication of cancer in patients undergoing DC-mediated anti-cancer therapy. In addition, current anti-cancer treatment regimens need to be carefully evaluated for their impact on DC function. Looking forward, the development of novel immunotherapeutic interventions for cancer should aim to enhance the migration, activation, maturation and/or function of tumor-associated DC to improve patient outcomes and exploit this critical immune cell type in future anti-cancer therapy.

## Figures and Tables

**Table 1:** The phenotype and functions of mouse and human DC subsets.

	Mouse DC Subsets		Human DC Subsets	
	Phenotype	Functions	Phenotype	Functions
**pDC**	CD45R, CD45RA, CD317	Anti-viral immunity Tolerance induction	CD123, CD303, CD304, CD45RA	Anti-viral immunity Tolerance induction
**cDC1**	CD8α or CD103, DEC205, Clec9A, XCR1	MHC I cross-presentation	CD141, DEC205, Clec9A, XCR1	MHC I cross-presentation MHC II presentation
**cDC2**	CD11b, Sirpα	MHC II presentation	CD1c, CD1a (skin), CD103 (mucosa)	MHC I cross-presentation MHC II presentation
**Inf-DC**	F4/80, Ly6C, CD64, FcεR1	MHC I cross-presentation MHC II presentation	CD1c, CD1a, FcεR1, CD14, CD206	MHC I cross-presentation Th17 induction
